# The use of trigger warnings on social media: a text analysis study of X

**DOI:** 10.1371/journal.pone.0322549

**Published:** 2025-04-30

**Authors:** Abigail Paradise Vit, Rami Puzis

**Affiliations:** 1 Department of Information System, Max Stern Yezreel Valley College, Israel; 2 Department of Software and Information Systems Engineering, Ben-Gurion University of the Negev, Be’er Sheva, Israel; Max Planck Institute for Solid State Research, GERMANY

## Abstract

Trigger warnings are placed at the beginning of potentially distressing content to provide individuals with the opportunity to avoid the content before exposure. Social media platforms use artificial intelligence to add automatic trigger warnings to certain images and videos, but are less commonly applied to textual content. This leaves the responsibility of adding trigger warnings to the authors, and a failure to do so may expose vulnerable users to sensitive or upsetting content. Due to limited research attention, there is a lack of understanding concerning what content is or is not considered triggering by social media users. To address this gap, we examine the use of trigger warnings in tweets on X, previously known as Twitter. We used a large language model (LLM) for zero-shot learning to identify the types of trigger warnings (e.g., violence, abuse) used in tweets and their prevalence. Additionally, we employed sentiment and emotion analysis to examine each trigger warning category, aiming to identify prevalent emotions and overall sentiment. Two datasets were collected: 48,168 tweets with explicit trigger warnings and 4,980,466 tweets with potentially triggering content. The analysis of the smaller dataset indicates that users have applied trigger warnings more frequently over the years and are applying them to a broader range of content categories than they did in the past. These findings may reflect users’ growing interest in creating a safe space and a supportive online community that is aware of diverse sensitivities among users. Despite these findings, our analysis of the larger dataset confirms a lack of trigger warnings in most potentially triggering content.

## Introduction

A trigger warning is a statement placed at the beginning of content (text, video, article, social media post, etc.) warning viewers or readers about the potentially distressing nature of the content [1,2]. Triggers are typically associated with various health conditions such as post-traumatic stress disorder (PTSD) or epilepsy [3]. PTSD is characterized by psychological and behavioral health conditions resulting from traumatic experiences [4,5]. People suffering from PTSD may respond to trauma triggers that make them feel as if a traumatic experience or event is recurring. There can be a variety of triggers, including a sound, sight, smell, thought, or other reminders of a past trauma [6]. Exposure to these triggers may result in extreme emotional reactions such as anxiety, panic, flashbacks, and other adverse reactions [3,7]. Depending on the individual and their unique experience and sensitivities, a wide range of topics can trigger unpleasant memories or reactions. These triggers may manifest when an individual is exposed to topics such as violence and sexual abuse [7,8]. Trigger warnings enable individuals to avoid exposure to distressing content [9].

Trigger warnings can be applied to a wide range of media, including video content such as television shows and movies, as well as print content such as books and magazines. Trigger warnings may also appear in games and apps, music, websites, radio programs, and research material [7]. In recent years, the use of trigger warnings has increased in a variety of fields (e.g., health, education, the arts) [7]. In 2014, trigger warnings made the headlines in higher education, when students at several US colleges demanded that trigger warnings be included in course syllabi and used in the classroom [10–15].

In this article, we focus on trigger warnings used on social media platforms [16]. Social media allows users to share a wide range of personal information; users can share their thoughts, experiences, and opinions. However, this freedom of expression can lead to the sharing of sensitive topics that may be upsetting to some individuals and potentially evoke distressing emotions or trigger past traumatic experiences [6]. A trigger warning can be added to potentially distressing content to warn users about it. While social networks automatically display trigger warnings for certain videos containing potential triggers (such as violence or sexually explicit content), this is generally not the case for textual posts (even textual posts that accompany a video or an image). It is typically the responsibility of the authors to decide whether to include trigger warnings in textual content.

In this study, we perform a comprehensive analysis of trigger warnings published on X (previously known as Twitter). To do this, 48,168 public tweets containing explicit trigger warnings (using the #triggerwarning hashtag) were collected and analyzed. We also collected and analyzed 4,980,466 tweets with content that may be triggering (some of these tweets include explicit trigger warnings). A large language model (LLM) for zero-shot learning was used to identify the trigger warning categories (such as violence or abuse). We found that the most common trigger warning categories were mental health, abuse, violence, stigma, and disturbing content.

Investigation of sentiment and emotions revealed that negative sentiment is strongly associated with trigger warning categories: fear is strongly associated with crime, sadness with mental health and death, and anger with stigma.

Overall, our analysis of the smaller dataset indicates that users have become increasingly aware of the types of content that may disturb other users. This trend facilitates a safe space and a supportive and sensitive online community. Yet, despite these positive trends, our analysis of the larger dataset highlights the lack of trigger warnings in most X content that could potentially cause distress. This indicates that significant disparity persists in the use of trigger warnings, leaving those suffering from PTSD and a wide range of sensitivities unprotected and exposed to triggering content.

To the best of our knowledge, this is the first work to perform a thorough data-driven analysis of trigger warnings on social media platforms.

## Related work

### Research on trigger warnings

Multiple studies have examined the use and impact of trigger warnings in educational settings [17,18]. Sanson et al. [2] examined whether presenting trigger warnings to college students before exposing them to negative course material affected their levels of negative affect, intrusive thoughts, and avoidance behaviors. The findings of their six experiments indicated that trigger warnings were neither meaningfully helpful nor harmful. In the medical education domain, Nolan and Roberts [14] conducted qualitative interviews with students to better understand their views on the functions, benefits, and drawbacks of using trigger warnings in the classroom. Students recognized warnings as a form of accommodation for trauma, but opinions on how and when they should be implemented varied. While these studies provide insights into the effectiveness of trigger warnings and their perception in a classroom setting, our study focuses on analyzing the use of trigger warnings on social networks.

Limited research has examined the efficacy and implications of trigger warning use on social media platforms. Hyland [19] examined people’s attitudes towards trigger warnings on social media using qualitative interviews, however only nine individuals were interviewed. Participants demonstrated positive attitudes towards the use of trigger warnings on social media; they also expressed concern regarding the misuse of trigger warnings for trivial content and emphasized that this misuse undermined the effectiveness of trigger warnings in general. Gupta [20] also examined social media users’ perceptions of trigger warnings through interviews with 15 regular social media users. The researchers concluded that users view trigger warnings positively but vary in terms of how they understand and use them.

In addition to user interviews, analysis of the actual user-generated content containing trigger warnings on social media platforms is required. Such analysis can provide insights into the prevalence of trigger warning use across social networks, the types and categories of triggers that are being warned about, temporal trends in their use, and other valuable insights. Nummenpää [21] examined the use of trigger tags as a form of censorship on Tumblr. The authors collected and analyzed 41,106 words describing emetophobia, agoraphobia, and arachnophobia. The study found that users are using meta-tagging conventions, reflecting a shift towards more cooperative censorship. The authors indicate that the evolving nature of one-to-many communication on social media leads to self-directed censorship and the need for regulation. Vlodder et al. [22] analyzed trigger warnings in 87 Facebook videos to explore how harmful the content presented in videos with trigger warnings on the social media platform is. The videos were analyzed using Braun and Clarke’s six-phase reflexive thematic analysis. According to the researchers, Facebook Watch provides opportunities for social actors to create and share social representations of harm through videos. The videos were found to be potentially impactful, persuasive, and extreme. The results highlighted the construction of social representations of harm through trigger warnings in social media videos. The authors recommended that further research should focus on assessing and developing guidelines for safe and inclusive social media platforms for both traumatized and non-traumatized individuals.

Another set of studies focused on establishing a structured taxonomy of trigger warnings. Charles et al. [7] presented a content warning typology based on electronic databases, journal tables of contents, website searches, citations, and expert consultation. In a review of 6,254 documents the authors identified 14 domains related to content warnings (listed from the highest frequency to the lowest): violence, sex, stigma, disturbing content, language, risky behavior, mental health, death, parental guidance, crime, abuse, socio-political, flashing lights, and objects. This study mainly focused on trigger warning guidelines and recommendations outlined in formal sources such as organizational policies and academic literature but did not analyze actual trigger warnings used by social network users.

Wiegmann et al. [23] viewed trigger warning assignment in terms of multi-label document classification and introduced a taxonomy of trigger warnings for textual content. They created the Webis Trigger Warning Corpus 2022, a dataset of HTML web pages, and mapped 41 million tags to 36 institutionally recommended trigger warnings using multi-label models such as SVM, XGBoost, and RoBERTa. The results show a low micro F1 score of about 0.50. The authors stated that their model may not transfer to other online content, such as news articles, websites, and social media posts. Stratta et al. [24] proposed automatic generation of trigger warnings for textual content. The authors developed a system that uses keyword identification, sentiment analysis, and online intervention principles to generate content warnings on the user’s end.

In our literature review, we found that most social media studies were qualitative and interview-based. In contrast, in this study, we perform a thorough data-driven analysis of trigger warnings in a large social media platform. To the best of our knowledge, we are the first to do so.

### Studies on ChatGPT-based classification

LLMs have demonstrated remarkable success in a variety of applications, including natural language processing (NLP) [25]. ChatGPT, which is built on LLM architecture, excels at understanding and executing complex text processing tasks [26] including information extraction, sentiment analysis, and text classification [27]. In addition, its flexibility in annotation tasks significantly reduces manual effort, streamlining the annotation process and accelerating NLP research [27].

ChatGPT has been used in several studies for a wide variety of classification tasks, including the text-davinci-003 model, which we also applied to our research. The text-davinci-003 model performs well across a variety of classification tasks. In stance detection [28], it effectively analyzed attitudes toward political candidates in social media posts, achieving an F1 score of 0.83 in multi-target stance detection and outperforming models such BERT. Similarly, in irony detection [29] text-davinci-003 outperformed traditional models, achieving an F1 score of 68.9% in binary irony detection.

In the legal domain, it was used for zero-shot semantic annotation of legal texts [30], where it demonstrated strong precision and recall, particularly in well-structured datasets, although it was less cost-effective than newer models like GPT-4. Additionally, text-davinci-003 has been successfully applied to job type classification task [31], achieving high recall of 95.6

In resume classification [32], it outperformed traditional approaches with an F1-score of 97.00%, demonstrating its effectiveness in talent acquisition tasks. Moreover, its application to domain-specific summarization [33] showed competitive results against state-of-the-art models like BART, particularly in less technical datasets. Lastly, text-davinci-003 proved to be a reliable tool for automated essay scoring in evaluating non-native English essays, capable of automatically evaluating and grading written essays [34].

Over time, more advanced LLMs continue to be released, and ChatGPT also includes more sophisticated versions that outperform text-davinci-003. Nevertheless, text-davinci-003 provided sufficient accuracy for this study, as confirmed by manual validation of a sample set of tweets (detailed in the Materials and methods section).

## Materials and methods

This research follows a standard analytical pipeline commonly used in social media research. The data collection process and analysis was approved by our institution’s review board: the Emek Yezreel College Ethical Review Board (approval number 2023-55 YVC EMEK). The collection and analysis method complied with the terms and conditions of the data source. Data collection from X was conducted using Tweepy, a Python wrapper for the official X API, ensuring adherence to X’s Terms of Service.

**Fig 1:**
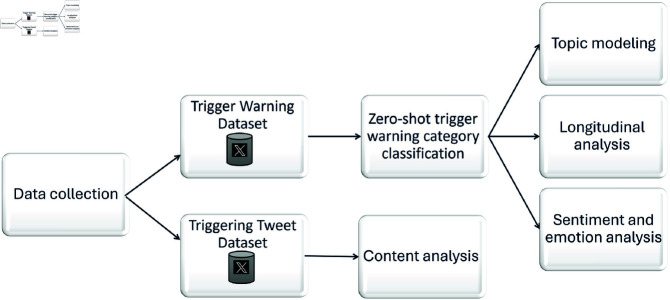
An overview of the analysis pipeline. The figure illustrates the methodology used in this study. The process begins with the collection of data, resulting in the creation of two datasets: the trigger warning dataset and the triggering tweet dataset. Various analyses were performed on these datasets.

The steps performed in our analysis are presented in Fig 1 and described in the subsections that follow. First, we collected the data and created two datasets: the trigger warning dataset and the triggering tweet dataset. The trigger warning dataset was split into tweets with textual content associated with one or more trigger warning categories and tweets without textual content associated with a trigger warning category. Then, topic modeling was performed for tweets without textual content associated with a trigger warning category. Sentiment and emotion analysis was performed for all tweets with textual content associated with one or more trigger warning categories. The triggering tweet dataset was analyzed to determine the prevalence of explicit trigger warnings in the tweets.

### Data collection

Our datasets were collected from the X social network by leveraging Tweepy, which is a Python library for accessing the X API. We collected tweets posted from November 26, 2009 through April 6, 2023.

For the first dataset, we collected tweets containing the #triggerwarning hashtag. A total of 48,168 public tweets were collected for this dataset, which we call the trigger warning dataset.

For the second dataset, we collected tweets with content that may be triggering or distressing to other users. A total of 11 hashtags were used to collect these tweets: #animalabuse, #domesticviolence, #childabuse, #pedophilia, #rape, #sexabuse, #stillbirth, #suicide, #selfharm, #babyloss, and #anorexia. A total of 4,980,466 tweets were collected for this dataset, which we call the triggering tweet dataset. These tweets may or may not include explicit trigger warnings.

The 11 hashtags were selected based on an analysis of the frequency of various trigger warning categories in the trigger warning dataset; a more detailed explanation about the hashtag selection will be provided in the triggering tweet dataset analyses subsection below.

### Trigger warning dataset analyses

#### Zero-shot trigger warning category classification

The objective of the tweet classification process was to accurately assign each tweet in the trigger warning dataset to its corresponding trigger warning category or categories (e.g., violence, abuse, self-harm), enabling proper labeling of the potentially disturbing content in the tweet content. Consequently, a tweet containing descriptions of a violent act would be assigned the violence trigger warning category, while a tweet discussing experiences with an eating disorder would be assigned the "eating disorder" trigger warning category.

In order to assign each tweet in the trigger warning dataset a trigger warning category or categories, an LLM for zero-shot classification was used. In zero-shot classification, the model classifies text into predefined categories without having seen any examples from the categories it is asked to identify during training [35]. The zero-shot classification model was used to divide the tweets into two groups: those for which the text in the tweet is associated with one or more of the trigger warning categories and those for which it is not; the groups are defined as follows:

1. **Tweets with textual content that is associated with one or more trigger warning categories:** Such tweets may trigger some users due to their sensitive or distressing content. The following are examples of such tweets:
  - Added URL #triggerwarning #cancer
  - it’s helped me to share this and hopefully, it can help others to read it #triggerwarning #miscarriage
  - i don’t appreciate animal abuse, even in jest. this is triggering me. alert #triggerwarning
2. **Tweets without textual content that is associated with a trigger warning category:** This category includes tweets that use a trigger warning but do not refer to a specific category of sensitive topics. Such tweets may still cause distress to certain individuals. In such cases, trigger warnings may be used satirically or as a rhetorical device to emphasize the content of the tweet. The following are examples of such tweets:
  - #triggerwarning don’t listen to the latest episode while flying.
  - Added image #triggerwarning #grossfood

We utilized the text-davinci-003 LLM for zero-shot classification. text-davinci-003 is a version of OpenAI’s GPT-3.5 model. This model was designed for high-quality text generation, zero-shot learning, and complex reasoning tasks. We used this classification model to determine whether or not the tweet was associated with any of the trigger warning categories.

For tweets that were associated with one or more trigger warning categories, the model assigned the associated trigger warning categories (e.g., violence, stillbirth, abuse), meaning that a single tweet could be classified into multiple categories.

To test the reliability of the model for the classification task, we randomly selected 500 tweets (250 tweets that were classified as not containing textual content associated with a trigger warning category and 250 tweets that were classified as containing textual content associated with a trigger warning category) for manual verification.

The configuration of text-davinci-003 included the following parameters: the classification produced by the model was limited to a maximum of 3,000 tokens, and only one text completion response was generated. In addition, a low temperature parameter of 0.1 was used to ensure a focused and deterministic response from the model.

The application of the text-davinci-003 model on the trigger warning dataset returned the subset of tweets containing textual content associated with a trigger warning category. We used this subset to examine the frequency distribution of different trigger warning categories in the tweets identified.

Using the text-davinci-003 model, specific classifications for each tweet were returned. We used the trigger warning guidelines published by Charles et al. [7] to determine the trigger warning category (or categories) for each classification. For example, if the model classified the tweet as animal cruelty, the tweet would fall under the trigger warning category of violence. The categories listed by the authors consist of several subcategories (e.g., mental health is a category, for which the subcategories include depression, anxiety, trauma, self-harm and suicide, and eating disorders).

#### Longitudinal analysis

As part of our investigation, we conducted a longitudinal analysis, which allowed us to identify trends in how trigger warnings have been used over time. We explored several aspects of trigger warning use over time on X. First, we examined the frequency of trigger warnings in tweets and the length of the tweets. We also calculated the number of distinct hashtags used in trigger warning tweets annually and the number of unique classifications identified by our trigger warning classification model. These classifications represent different categories or subcategories of trigger warnings derived from tweet content.

#### Sentiment and emotion analyses

We also assessed the sentiments and emotions expressed in the tweets to gain insight into the emotional context associated with the different trigger warning categories.

Sentiment analysis was performed using a RoBERTa-based model that was trained on 124 million tweets and fine-tuned using the TweetEval benchmark [36]. This model is available in the HuggingFace repository under the name cardiffnlp/twitter-roberta-base-sentiment- latest.

A fine-tuned checkpoint of the DistilRoBERTa-base [37] was used to analyze the six basic emotions (fear, anger, disgust, joy, sadness, surprise) suggested by Ekman [38]. The model, which was trained on six diverse datasets, is available in the HuggingFace repository under the name j-hartmann/emotion-english-distilroberta-base on HuggingFace.

#### Topic modeling

To gain deeper understanding about the tweets in the trigger warning dataset that were categorized in the tweet classification process described in the previous subsection as not containing textual content associated with trigger warning categories, BERTopic [39] was used for topic modeling (only for these tweets); this allowed us to identify the topics of tweets that did not contain textual content associated with a trigger warning category, as well as the topics of tweets where trigger warnings were redundant or excessive.

In BERTopic, topic representations are created in a six-step process, and different algorithms and models can be used as inputs at various stages of the process.

The following configurations were used across the following steps:

1. Embedded tweets—Our tweets are converted into numerical representations, using the all-MiniLM-L6-v2 sentence transformer [40].
2. Dimensionality reduction—The dimensionality of the embedded tweets is reduced using Uniform Manifold Approximation and Projection (UMAP) [41].
3. Cluster tweets—Tweets are grouped into clusters using HDBSCAN [42], a density-based clustering technique.
4. Bag-of-words—Using this bag-of-words representation, the frequency of each word in each cluster is determined on a cluster level (as opposed to a tweet level).
5. Topic representation—In order to create topic representations of tweets that were clustered within the same cluster, BERTopic uses c-TF-IDF (class-based term frequency-inverse document frequency); an enhancement of TF-IDF, c-TF-IDF allows representation at the cluster/topic level. Equation 1 presents the calculation as described in [39], where the c-TF-IDF weight for a term *t* in a cluster *c* is computed as the product of *tf_t,c_* (the term frequency of *t* in cluster *c*) and the logarithm of the ratio of *A* (the average number of words per cluster) to t*f_t_* (the total frequency of *t* across all clusters).
  $$\begin{array}{ccc} W_{t,c}=tf_{t,c}\cdot \log\left(1+\frac{A}{tf_{t}}\right) & & \end{array}$$ (1)
6. Outlier reduction—In step 3, HDBSCAN identifies tweets that are outliers and do not fall in any of the topics created. In this step, we reduce the number of tweets classified as outliers by calculating the c-TF-IDF representation for each outlying tweet and finding the best matching c-TF-IDF topic representation based on the cosine similarity.

### Triggering tweet dataset analyses

#### Content analysis

We also investigated the prevalence of trigger warnings in tweets with textual content associated with one or more trigger warning categories in the triggering tweet dataset which was collected using 11 hashtags. In our analysis of the frequency of various trigger warning categories in the trigger warning dataset, the five most common categories were identified. A total of 11 random subcategories were selected from these top categories. We then searched the hashtag in the X social media platform for each subcategory.

The textual content of each tweet was analyzed to determine whether it contained an explicit trigger warning by searching for the following keywords: content warning, trigger warning, triggerwarning, #triggerwarning, and #tw.

For each of the hashtags, we counted the total number of tweets containing the hashtag and how many of those tweets contained an explicit trigger warning. We calculated the percentage by dividing the number of tweets with trigger warnings by the total number of tweets for that hashtag; for each hashtag, the value represents the percentage of tweets containing the hashtag that included an explicit trigger.

This enabled us to quantify and compare the frequency at which trigger warnings are used across different hashtags related to content that may be triggering or distressing to other users.

## Results

### Results for trigger warning dataset

We used the text-davinci-003 classification model to divide the tweets into two groups: those for which the text in the tweet is associated with one or more of the trigger warning categories and those for which it is not. A total of 36,994 tweets were classified as not containing textual content associated with a trigger warning category, while 11,174 tweets were classified as textual content associated with one or more trigger warning categories.

To test the reliability of the model for the classification task, we randomly selected 500 tweets for manual verification and found that 96.65% of the tweets were correctly classified by the model. This indicates that the model was capable of correctly categorizing the tweets with a high degree of accuracy.

#### Analysis of tweets with textual content associated with one or more trigger warning categories.

##### Results of longitudinal analysis

**Fig 2:**
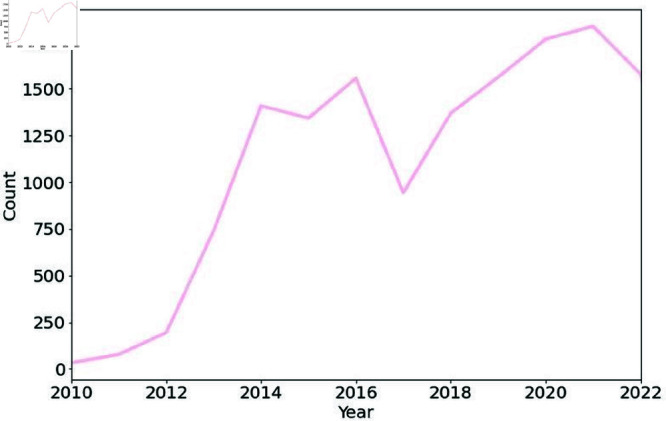
Frequency of tweets with textual content associated with one or more trigger warning categories over the years. Adoption of trigger warnings on X generally increased until 2016, followed by a decline in 2017 and then began to increase again.

First, we examined the use of trigger warnings over the years. The results of our longitudinal analysis are presented in Fig 2, which shows that the adoption of trigger warnings on X has increased over time. A significant increase in their use was observed in 2014. The use of trigger warnings continued to grow until 2016. In 2017, there was a decrease in their use; this was followed by an upward trend that continued until 2021.

**Fig 3:**
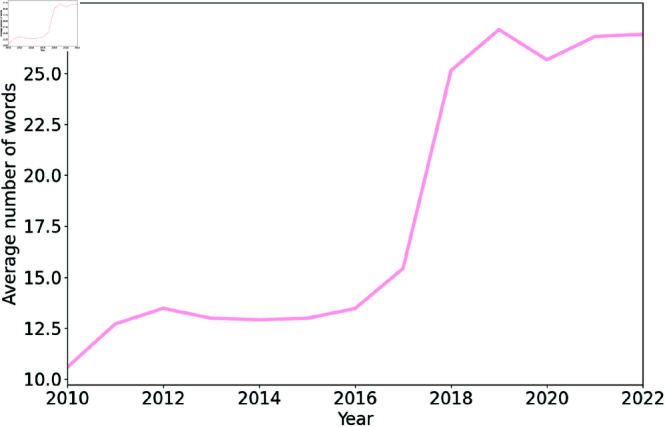
Average number of words in tweets with textual content associated with one or more trigger warning categories over the years. The average number of words in tweets has increased over time.

To gain further insight on tweets containing textual content associated with a trigger warning, we compared the length of these tweets over time. Fig 3 presents the average number of words in textual tweets over the years. As can be seen, the average number of words has increased over time. A significant increase in the average number of words was observed in 2018; that year the average number of words was more than twice as high as in 2016. This increase coincided with an increase in the maximum number of characters per tweet (from 140 to 280) that year. It is possible that this gave users more space to express themselves and the ability to devote characters to trigger warnings when necessary.

**Fig 4:**
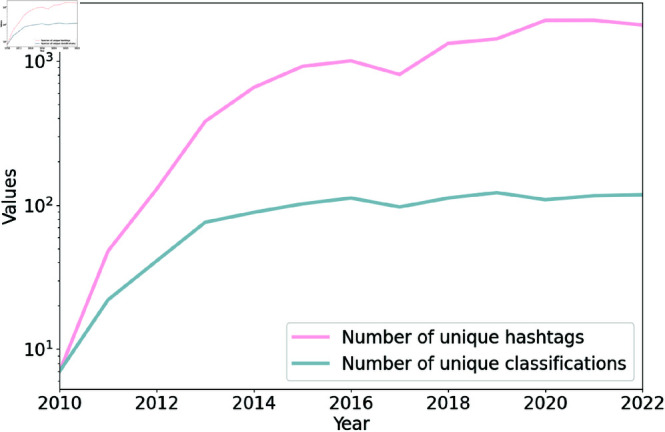
Number of unique classifications and hashtags per year. The number of unique hashtags refers to the total count of distinct hashtags used in tweets each year. The number of unique classifications refers to the total count of distinct trigger warning categories or subcategories derived from the content of tweets each year.

Fig 4 presents the number of distinct hashtags used in tweets per year and the number of unique classifications identified by the classification model each year. The classifications identified represent the trigger warning categories or subcategories derived from the content of the tweets. The y-axis is presented on a logarithmic scale. Each year the number of unique hashtags used in tweets was larger than the number of unique classifications provided by the model. The number of hashtags consistently exceeds the number of classifications, suggesting that many hashtags are unrelated to trigger warning categories.

**Fig 5:**
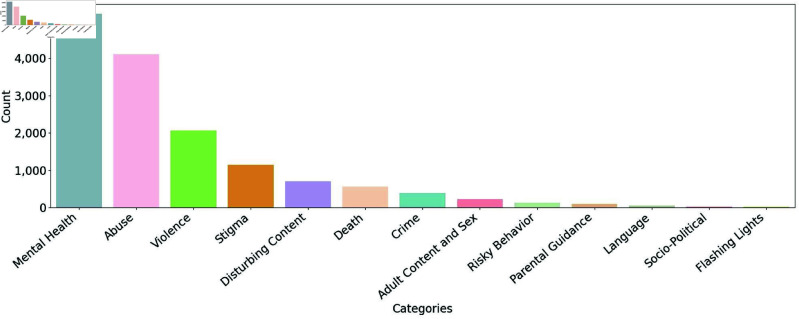
Number of tweets per trigger warning category. Mental health, abuse, violence, stigma, and disturbing content are the most frequent categories.

In Fig 5, we present the main trigger warning categories provided by the model for the tweets in the trigger warning dataset. As can be seen, mental health, abuse, violence, stigma, and disturbing content are the most frequent categories.

We calculated the c-TF-IDF value to determine the most representative words for each trigger warning category (meaning the words that are most relevant to each category). The calculation was based on Equation 1. As a result, we were able to determine the most significant words in each category. S1 Appendix contains the top-1,000 words with the highest c-TF-IDF for each trigger warning category. As can be seen, for each category, the significant words are closely related to the topic at hand. For example, violence category, includes terms related directly to acts of violence (e.g., domestic violence, gore, women, shooting, and gun). Similarly, for the category of abuse, the words are indicative of abuse and its discourse (e.g., rape, metoo, sexual assault, survivor, and victim).

##### Results of sentiment and emotion analysis

We examined the sentiments and emotions in the tweets associated with the different trigger warning categories. We did not include the emotion and sentiment of trigger warning categories with less than 300 tweets in our analysis. This was done in order to ensure that we only considered categories for which there was sufficient data to accurately reflect the sentiment and emotion, rather than relying on a few tweets that may not accurately reflect a category’s true sentiment and emotion.

**Fig 6:**
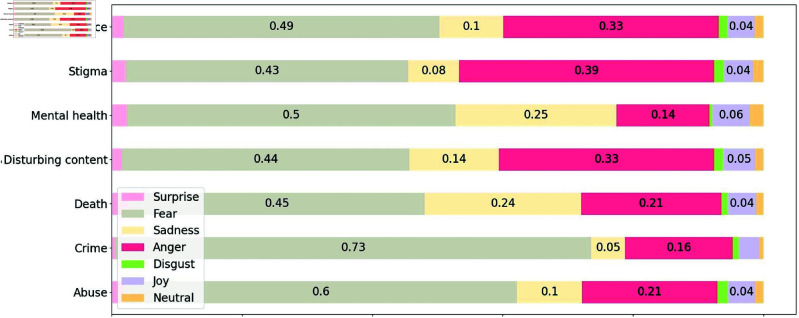
Emotions (fear, surprise, sadness, anger, disgust, joy, and neutral) associated with the top-7 trigger warning categories. Sadness, fear and anger are the dominant negative emotions in these categories. The categories with the highest emotion scores for sadness are death and mental health.

Fig 6 presents the average emotion score for seven emotions (fear, surprise, sadness, anger, disgust, joy, and neurtal) for the top-7 trigger warning categories that appeared most frequently in Fig 5. The dominant negative emotions in the categories are fear, sadness and anger. Death and mental health are the categories with the highest emotion scores for sadness. Out of all the trigger warning categories examined, the stigma category had the highest average emotion score for anger; topics covered in the stigma category include racism, and the tweets are more heated and angry. For the crime category, the emotion of fear obtained the highest emotion score. This category included tweets covering topics such as murder, kidnapping, lynching, human trafficking, and bloodshed.

**Fig 7:**
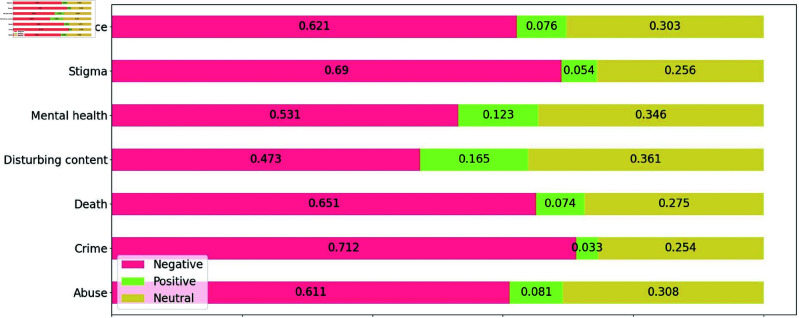
Sentiment (negative, positive, and neutral) associated with the top-7 trigger warning categories. Across all categories, negative sentiment was predominant, while positive sentiment was slightly higher in the categories of disturbing content and mental health.

In Fig 7, the average sentiment scores of the top-7 trigger warning categories are presented. As can be seen, negative sentiment was dominant across all categories; positive sentiment was slightly high in the disturbing content and mental health categories (0.165 and 0.123 respectively). As noted in the dataset analyses subsections of the methods section, each category contains several subcategories. Therefore, to determine which subcategories (specific subjects) had a higher level of positive sentiment, we performed further analysis of the subcategories of disturbing content and mental health categories.

Rather than calculating the average sentiment at the broad category level, we analyzed sentiment at the subcategory level. Obtaining the average sentiment score for each subcategory will deepen our understanding of the sentiment across various topics within each category.

We started by examining the sentiment of each subcategory in the mental health category. Table 1 present the four subcategories with the highest positive sentiment scores in the mental health category. We found that the subcategories of OCD (obsessive-compulsive disorder), panic attacks, eating disorders, and general mental health have higher positive sentiment score than the other subcategories. We then manually examined the tweets classified as belonging to these subcategories to determine why these subcategories received high positive sentiment scores.

**Table 1:** Top-4 subcategories with the highest positive scores in the mental health and category.

Category	Subcategory	Positive sentiment score
Mental health	OCD	0.264
Mental health	Eating disorders	0.178
Mental health	Panic attacks	0.156
Mental health	General mental health	0.153

The following table lists the four subcategories within the mental health category with the highest positive sentiment scores. OCD, panic attacks, eating disorders, and general mental health have higher positive sentiment scores than other subcategories.

Based on our review of the tweets related to general mental health and OCD, it seems like the tweets are more positive, because they provide a positive recommendation about certain content while also mentioning the content’s trigger warning category. The following is an example of such a tweet:

- #triggerwarning: panic attack. proud about myself. this morning a colleague of mine had a panic attack due to some stupid mistake at work. i was able to manage her panic attack, i was able to comport her, i was able to make her feel a little bit better.

In most tweets related to panic attacks, the panic attack is described, as well as how it was overcome. In the case of eating disorders, most tweets are related to users who are proud of themselves for losing weight or not eating. Many of these tweets include hashtags associated with eating disorders [43] such as #proana, #thinspo, and #promia. Prior work has found that these types of hashtags encourage people with eating disorders to continue their destructive behavior [43–45]. Being aware of this type of content and avoiding it is critical for those with eating disorders. The following are examples of such tweets:

- my hands look so ana i love it #triggerwarning #ana #bdd #binge
- Imagine how much more amazing you’d look if your body looked like this #thinspo #bonespo #meanspo #edtwt #triggerwarning #ed #hipspo #armspo #legspo

We also examined the sentiment in the subcategories of the disturbing content category. Table 2 presents the two subcategories with the highest positive sentiment scores in this category. As can be seen in the table, the human body and functions subcategory has a higher positive sentiment score than other subcategories. Tweets in this subcategory were related to content about makeup of blood. While this is potentially disturbing content, a positive description of the outcome was also provided in the tweets. The following is an example of such a tweet:.

- I got really proud of this one! #illustration #digitalart #triggerwarning #Blood #Dark

**Table 2:** Top-2 subcategories with the highest positive sentiment scores in the disturbing content category.

Category	Subcategory	Positive sentiment score
Disturbing content	Human body and functions	0.225
Disturbing content	Horror and scary content	0.118

The following table lists the two subcategories within the disturbing content category with the highest positive sentiment scores. The human body and functions and horror and scary content have higher positive sentiment scores than other subcategories.

#### Analysis of tweets without textual content associated with a trigger warning category

**Results of topic modeling.** Of the 48,168 tweets in the trigger warning dataset, 36,994 tweets were classified as tweets without textual content associated with trigger warning categories by the text-davinci-003 model. The tweets were then categorized using the BERTopic model.

There were 41 topics with a coherence of 0.717. After combining five topics related to the same television show into one topic (trigger warning show), the coherence was 0.725 with 37 topics.

The seven most frequent topics are listed in Table 3, which presents the number of tweets for each of the top-7 topics along with the topic name, which consists of the words with the highest c-TF-IDF for that topic.

**Table 3:** Top-7 topics.

Number of tweets	Topic number and name
6,459	0_to_you_this_the
3,218	1_based_real_attn_mon
2,198	2_read_book_story_reading
1,830	3_women_men_feminism_the
1,808	4_trump_maga_debatenight_the
1,722	5_mike_killer_killermike_mario
1,463	6_episode_show_season_of

Table 3 presents the seven most frequent topics with the number of tweets for each of the top-7 topics along with the topic name, which consists of the words with the highest c-TF-IDF for that topic.

In our analysis, we found that for many (14) topics, users did not specify which trigger warning categories they were referring to but simply indicated that the content may contain triggers (such as a movie, podcast, song, video). Below are some examples of such tweets:

- 6_episode_show_season_of - #triggerwarning don’t listen to the latest episode while flying.
- 16_watch_watching_to_you - #triggerwarning #13reasonswhy is a show parents should be watching with their kids. not kids alone. there is so much to process
- 22_video_youtube_videos_the - #triggerwarning - this truly saddens me. this video is definitely worth a watch.
- 23_thread_oc_interesting_red - #triggerwarning on this entire thread.

We also identified an additional group of topics related to a content called "Trigger Warning". For example, there were tweets about a book called "Trigger Warning" in topic number 2. A TV show on Netflix called "Trigger Warning" was discussed in topic 5. Below are some examples of such tweets:

- 2_read_book_story_reading- it came! so excited. that’s my sunday all sorted #neilgaiman #triggerwarning
- 5_mike_killer_killermike_mario- #triggerwarning #killermike episode 1, #buyingblack review

In addition, we found that there are topics that contain trigger warnings satirically, humorously, or critically; for example, topic 4 contains such tweets regarding Donald Trump, with both sides (those in favor of Trump and those opposing him) including trigger warnings in their tweets. In this case, the trigger warning is not necessarily used to signal distressing material, but rather to convey a sense of irony, humor, or criticism. For example, users might use trigger warnings critically when discussing Trump to suggest that the content may be deemed provocative or contentious. Another example is topic 14, which relates to food. In this case, trigger warnings are applied to content that is not typically distressing or triggering and are used in a humorous, satirical, or exaggerated manner to highlight and draw attention to things like pizza or vegan food. Some examples of these types of tweets are provided below:

- 4_trump_maga_debatenight_the - #triggerwarning: the following tweet may come as a shock to those on the left: trump voters did not need #putin to persuade them
- 4_trump_maga_debatenight_the - i forgot to add my #triggerwarning, my bad. #comeyday #impeachtrump #trumplies #obstructionofjustice
- 4_trump_maga_debatenight_the - i have the perfect way to trigger liberals. i went to two trump rallies and didn’t get covid #triggerwarning #liberaltears
- 14_food_vegan_pizza_eat - #triggerwarning #pizza

Socio-cultural topics are included in the last group of topics. Topic 29 refers to the term "snowflakes" [46–48], an insult intended to mock those who are excessively sensitive or easily offended (particularly in political and social contexts), often delivered from the right to the left targeting liberals.

The trigger warning and safespace hashtags are also used by these users. A safe space is a space that is intended to be free from discrimination, harassment, or any other form of harm [49,50]. The term appears mainly in topic 17, but it also appears in other topics, and we found that both those who support a safe space and trigger warnings and those who reject them use the safespace hashtag.

Topic 15 relates to hashtags associated with social justice warriors (SJWs) and cultural Marxism. Social media is being used by activists and movements for social justice, speaking out on issues such as racism, harassment, and violence. In 2016, social justice movements gained widespread attention on social media in response to events such as the Black Lives Matter movement, and discussions about police brutality, the US presidential election, debates about immigration policies, and LGBTQ+ rights [51–54]. A surge of tweets related to social justice issues was sparked by these movements and events. In most cases, the hashtags associated with SJWs are used in a pejorative manner to describe individuals who advocate for social justice and equality [55,56].

The term cultural Marxism describes a theory that posits that social and cultural changes are influenced by Marxist ideas [57]. The term is often used in a pejorative manner by those who consider Marxism to be a threat to traditional cultural values.

Topic 7 is related to the #freespeech hashtag, whereas topic 25 is related to the antifreespeech hashtag. These hashtags are often used to refer to debates regarding free speech, censorship, hate speech, and online harassment. The #antifreespeech hashtag [58,59] is typically employed by users who feel that their right to free speech is being suppressed, especially in cases where their views are regarded as controversial or offensive.

Topic 32 pertains to the #politicalcorrectness hashtag [60]. It is often used to refer to a perceived excessive emphasis on avoiding language or behavior that might be considered discriminatory or offensive, particularly in relation to race, gender, and sexuality issues, by users that think that the emphasis on political correctness has gone too far and limits their freedom of expression.

Some examples of tweets related to those topics are provided below:

- 7_students_college_university_freespeech - dehumanizing others through censorship does not befit the academy, but the pigpen #triggerwarning #censorship #tcot
- 15_sjw_culturalmarxism_thoughtpolice_socialjusticewarriors - turns out some people don’t like to be questioned? freedom of speech is never free. #sjw #fckter #triggerwarning #sjwlogic #gendernomics
- 17_safespace_safe_space_safespaces - do you need a safe space little snowflake? generation #ed. #safespace #sjw #socialjusticewarriors Now the surveys are hurting people, too! #SafeSpace #TriggerWarning #EndTimes
- 25_ed_antifreespeech_safespace_ing - leftist #losers #antifreespeech #politicalcorrectness #safespace ed #triggerwarning
- 29_snowflakes_snowflake_safespace_along - poor little #snowflake melting under the ’blazing cameras’ pence needs his #safespace #TriggerWarning intelligent #poc 32_libby_politicalcorrectness_dontgofulllibtard_stupiddemocrats - #triggerwarning , i’m sure canada doesn’t want you #liberal fucktards either #feelthebern

### Results for triggering tweet dataset

#### Results of content analysis

The purpose of this analysis was to determine whether users proactively incorporate trigger warnings when discussing potentially triggering topics.

For each hashtag, Table 4 presents the number of tweets containing the hashtag, along with the number and percentage of tweets containing a trigger warning. As can be seen in the table, trigger warnings are not prevalent in the analyzed tweets. In fact, they are quite rare, appearing in less than 1% of all hashtags, indicating that the use of trigger warnings in content that may trigger other users does not appear to be widespread.

**Table 4:** Hashtag analysis summary.

Hashtag	Number of tweets	Number of tweets with trigger warning	Percentage of tweets with trigger warning
#selfharm	111,488	840	0.75%
#babyloss	84,354	618	0.73%
#sexabuse	43,662	100	0.23%
#domesticviolence	964,037	1,654	0.17%
#rape	1,052,505	1,694	0.16%
#anorexia	216,246	289	0.13%
#suicide	1,382,154	1,542	0.11%
#stillbirth	53,615	56	0.10%
#pedophilia	55,190	31	0.06%
#childabuse	786,592	383	0.05%
#animalabuse	230,623	104	0.05%

The table presents the hashtag, the number of tweets containing the hashtag, and the number and percentage of tweets including a trigger warning. Most tweets that contain potentially triggering content arising from the selected hashtags do not include trigger warnings.

**Fig 8:**
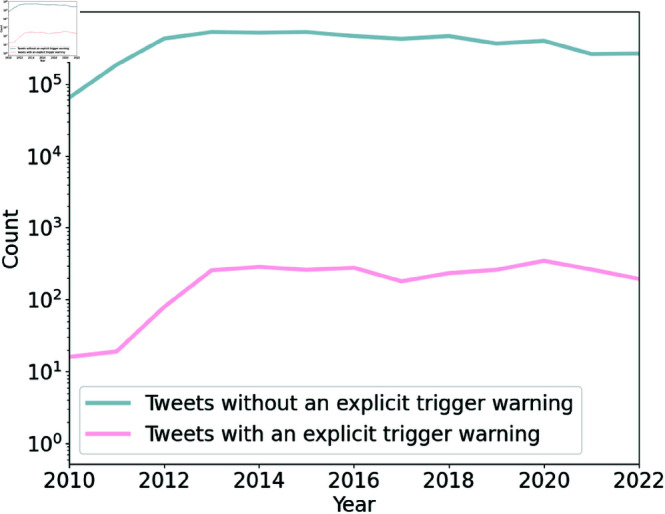
Frequency of tweets with and without an explicit trigger warning over the years. A comparison between tweets that include a trigger warning and those that do not contain a trigger warning over time. The y-axis is presented on a logarithmic scale. The use of trigger warnings has increased over time, but they are not explicitly included in the majority of tweets that contain triggering content.

Fig 8 provides a comparison between tweets that include a trigger warning and those that do not contain a trigger warning over time. The y-axis is presented on a logarithmic scale. As can be seen, although trigger warnings have been used more frequently over the years, they are not explicitly included in the majority of tweets that contain triggering content. Examples of such tweets include:

- I sincerely hope that you heard the voices of grieving parents; families who have experienced #stillbirth in #WashingtonState because we are not alone. Every state has its own number; rate of loss. Please Senator, help us help others, in memory of our babies
- 6 months after the DAV Public School sexual assault of a 4 y.o girl, the fast-track special court for #POCSO sentenced Beemana Rajani Kumar to 20 years in jail fine of Rs 5,000 #indianrapists #incredibleindia #maleviolence #rape #hyderabad #sexnotgender
- BBC News—was born from rape—but I wont let it define me .#rape #sexualviolence URL

## Discussion

### Insights from longitudinal analysis

The results showed that the adoption of trigger warnings has generally increased over time. The use of trigger warnings has been influenced by debate and discussion about their effectiveness and necessity in blogs, forums, and articles, among the academic community, and by the media; in 2012-2013, the debate on trigger warnings started to gain attention in online blogging when feminist bloggers began raising awareness about it [6]. The feminist blogging community provides trigger warnings for content related to war, abuse, self-harm, mental health, abuse, eating disorders, and body shaming [61,62]. In 2013, on the popular Shakesville blog, Ruxandra Looft, a lecturer at Iowa State University, argued that trigger warnings have a critical role to play in the classroom [63]. A significant increase in tweets with trigger warnings was observed in 2014. This may be due to the fact that discussion on trigger warnings has continued in educational settings. US Students have been requesting content warnings at the beginning of lectures discussing material that might trigger negative emotional reactions. Colleges and universities began implementing trigger warnings in their curriculum to alert students of potentially distressing content in their curriculum [64].

Furthermore, the results included a yearly count of the unique hashtags and classifications identified. The number of hashtags consistently exceeded the number of classifications. This finding suggests that not all hashtags used in tweets fall under trigger warning categories or subcategories. There are many hashtags that are not related to potentially sensitive content but rather relate to general topics, locations, events, or other subjects.

Despite the difference in the number of hashtags and classifications, an upward trend can be seen. Based on these findings regarding the trigger warning categories identified by the model and distinct hashtag use, we can conclude that the range of potentially sensitive topics being discussed in tweets has increased from year to year. Users are introducing new categories of content that may require trigger warnings that were not prevalent or considered sensitive topics in the past. This may indicate that users are increasingly aware of the diversity of sensitivity, trauma, and experiences among other users and are using trigger warnings more frequently when dealing with a range of sensitive topics in order to promote a safe and supportive online community.

Regarding the trigger warning categories identified by the model, the most frequent categories were mental health, abuse, violence, stigma, and disturbing content are the most frequent categories. According to the article that presented the taxonomy of trigger warnings, violence, sex, stigma, disturbing content, and language were the main categories [7]. The difference in the categories can be attributed to the fact that in Charles et al. [7], they focused on content published in academic journals, technical reports, books, and web pages, as opposed to user-generated content on social media platforms. Some categories, such as violence or sex, may be more prevalent in certain domains (such as entertainment or news) than in others. On the other hand, social media is known for the prevalence of discourse on mental health [65,66] and sexual abuse [67]. Statistics published by the World Health Organization indicate that approximately 3.8% of the population suffers from depression, and approximately 700,000 people commit suicide each year. The number of tweets about mental health is increasing in correlation with the total number of tweets on X [65,66]. Moreover, in recent years, social media has increasingly served as a platform for survivors of sexual abuse to share their stories [67]; social media users often disclose their personal experiences with the #metoo hashtag. This term was coined in 2016 by a group of activists to raise awareness about sexual abuse. It became viral in October 2017, following allegations of sexual misconduct in the Hollywood community [68]. Increased awareness of sexual abuse on social networks has led to a significant amount of sexual abuse-related content in tweets with trigger warnings.

### Insights from sentiment and emotion analysis

The dominant emotions observed in tweets with trigger warnings were fear, sadness, and anger. This is not surprising given that trigger warnings are intended to alert users to content that may frighten or upset others. Sadness being highest in the death and mental health categories aligns with themes of loss and emotional struggles. Similarly, anger’s prominence in the stigma category is tied to topics like racism, which may evoke heated discussions. Fear was notably high in the crime category, aligning with its emphasis on distressing topics such as murder, kidnapping, and human trafficking.

The prevalence of negative sentiment across categories also emphasizes trigger warning content’s sensitive nature. The higher positive sentiment in disturbing content and mental health categories expressed positive accomplishments or recommendations despite being associated with triggering content. For example, tweets in the eating disorders subcategory contained positive language related to harmful behaviors, such as weight loss success or restrictive eating.

After thoroughly examining the categories and subcategories that had higher positive sentiment scores and after reviewing the relevant tweets, we found that there are three types of tweets with a higher positive sentiment score:

1. **Inspiring and supportive tweets:** Tweets intended to empower or encourage others. Along with support and resources, tweets may contain distressing content. For example, a tweet discussing a personal experience of overcoming trauma may contain distressing details, while also conveying a message of resilience, hope, and recovery. This can result in tweets with mixed emotions and sentiments, including positive sentiments and emotions.
2. **Recommendation tweets:** Tweets that contain positive recommendations regarding content such as books, TV shows, podcasts, and songs. In this case, a trigger warning regarding the recommended content is included in the tweets, but the positive recommendation is considered positive sentiment.
3. **Dangerous or destructive tweets:** Tweets that promote dangerous or destructive actions, ideas, or behaviors in a positive and cheerful manner. The danger of these tweets lies in the way they present harmful ideas by making them appear more appealing and natural. Tweets of this sort may encourage other users to engage in destructive or harmful behavior. Despite the potential harm they can cause, such tweets have a higher positive sentiment score due to their pleasant nature.

### Insights from topic modeling analysis

The results of the topic modeling analysis for tweets without textual content explicitly linked to trigger warning categories provide insights into the diverse ways users interact with and utilize trigger warnings across different contexts. One observation is that trigger warnings are not always used as protective measures but may be repurposed as rhetorical tools to convey irony, humor, or critique. For example, tweets referencing political figures like Donald Trump or food-related topics demonstrate how trigger warnings can serve as a means of satire or social commentary, rather than solely signaling potentially distressing content.

Socio-cultural topics, including debates about free speech, political correctness [60], and social justice [55,56], further emphasize how trigger warnings intersect with broader societal issues. Hashtags like #freespeech and #antifreespeech [58,59] highlight ongoing discussions about censorship and the balance between freedom of expression and sensitivity.

These findings reveal that trigger warnings are again extended beyond their original intent to signal distressing content. They serve as a versatile communication tool, cultural commentary, and political discourse. However, this expanded use underscores the need for accurately interpreting their meaning and purpose.

### Insights from content analysis

The content analysis highlights a significant gap in the proactive use of trigger warnings in tweets discussing potentially distressing topics. Despite the growing awareness of trigger warnings, they remain rare, appearing in less than 1% of tweets across the analyzed hashtags. While hashtags like #suicide or #rape may signal potentially triggering content, they do not explicitly function as trigger warnings, leaving users to infer the nature of the content. The absence of explicit trigger warnings suggests that users may perceive social networks as unsafe environments, where potentially distressing content is readily accessible. This lack of protection leaves individuals with PTSD and other sensitivities vulnerable to exposure to triggering material. It is recommended that social media platforms, browser extensions, and third-party applications enhance their content warning mechanisms by providing customizable content filters, which can contribute to a safer online environment for these users.

### Limitation

Our study has some limitations. The first limitation is that the study relied solely on data from platform X, which has its own unique characteristics and distinct audience. As a result, the insights may be limited to this platform alone. To gain a more comprehensive understanding, it is necessary to expand the research to include data from additional social networks.

Second, our findings depend on the classification model used to identify trigger warning categories, which may introduce biases. Specifically, we employed zero-shot classification using an LLM without fine-tuning, meaning that the model’s classifications are influenced by the data it was originally trained on. To verify the model’s reliability, we conducted a manual review of a sample of classifications and assessed the consistency and accuracy of the model’s responses. Furthermore, we applied topic modeling to tweets classified as not containing trigger warnings categories. If the LLM had been mostly inaccurate, we would have expected topics related to trigger warnings to emerge. However, the generated topics were unrelated to such categories, suggesting the classification was mostly reliable.

Despite these efforts, this approach does not entirely eliminate the possibility of misclassification.

## Conclusions

In this study, valuable insights were gained regarding the use and prevalence of trigger warnings on the X social media platform. Tweets from 2009 through 2023 were collected. Mental health, abuse, violence, stigma, and disturbing content were found to be the most common trigger warning categories utilized by users. We also investigated the sentiments and emotions associated with the various trigger warning categories, and our analysis showed that there is a high level of negative sentiment associated with trigger warning categories. More specifically, we found that the sentiment of fear is associated with crime, sadness is associated with mental health and death, and anger is associated with stigma.

We also found that positive sentiment was slightly higher in the disturbing content and mental health categories. Based on our review of the tweets related to these categories, we identified three types of tweets with higher positive sentiment: inspiring and supportive tweets aimed at empowering or encouraging others, often including support and resources despite distressing content; recommendation tweets that contain trigger warnings but also offer positive reviews of content such as TV shows, podcasts; and dangerous or destructive tweets that promote harmful actions, ideas, or behaviors in a positive and cheerful manner.

Our analysis revealed an upward trend in the use of explicit trigger warnings over time. In recent years, trigger warnings appear to have been used by users more frequently and across a wider range of content categories, indicating increased awareness of diverse sensitivities and potentially distressing material. However, it is important to note that despite the observed trends, the majority of content that could be triggering lacks explicit trigger warnings. It is our hope that our study raises awareness of this gap, particularly among users who do not wish to be exposed to distressing online content.

As part of our future research, we plan to investigate the use of trigger warnings on other social media platforms such as Facebook and Instagram.

## Supporting information

S1 Appendix The most representative words for the trigger warning category. CSV file of top c-TF-IDF words for the trigger warning categories.
(CSV)
